# Anti-Tumor Necrosis Factor Receptor 2 Antibody Combined With Anti-PD-L1 Therapy Exerts Robust Antitumor Effects in Breast Cancer

**DOI:** 10.3389/fcell.2021.720472

**Published:** 2021-11-25

**Authors:** Qiang Fu, Qian Shen, Jin Tong, Liu Huang, Yi Cheng, Wei Zhong

**Affiliations:** ^1^Department of Oncology, Tongji Hospital, Tongji Medical College, Huazhong University of Science and Technology, Wuhan, China; ^2^Department of Breast Cancer, Hubei Cancer Hospital, Huazhong University of Science and Technology, Wuhan, China; ^3^Department of Peripherally Inserted Central Catheter (PICC), Tongji Hospital, Tongji Medical College, Huazhong University of Science and Technology, Wuhan, China

**Keywords:** TNFR2, breast cancer, TNFR2 antagonist, anti-PD-L1 therapy, exert robust anti-tumor

## Abstract

Breast cancer is a leading type of malignant tumor in women; however, the immunotherapy in breast cancer is still underappreciated. In this study, we demonstrated that tumor necrosis factor receptor 2 (TNFR2) is highly expressed in both breast tumor tissue and tumor-infiltrating immunosuppressive CD4^+^Foxp3^+^ regulatory T cells (Tregs). We found that TNFR2 antagonistic antibody reduced Foxp3 expression and the proliferation of Tregs and impaired the inhibitory effect of Tregs on CD4^+^CD25^–^ effector T (Teff) cells in a dose-dependent manner. The treatment of anti-TNFR2 antibody not only inhibited the proliferation of breast tumor cells *in vitro* but also suppressed the tumorigenesis of murine mammary carcinoma 4T1 cells *in vivo*. Mice recovered from tumor growth also developed 4T1-specific immunity. Furthermore, we demonstrated that anti-TNFR2 antibody in combination with anti-PD-L1 exhibited augmented antitumor effects than monotherapy. Anti-TNFR2 treatment also tended to increase the expression of proinflammatory cytokines in tumor tissues. In conclusion, our study suggests that TNFR2 antagonist could potentially offer a clinical benefit as a single agent or in combination with immune checkpoint blockade treatment for breast cancer immunotherapy.

## Introduction

Breast cancer is a major threat to the health of women in the world. In 2020, it accounts for 30% of all new cancer cases in the United States, and more than 41,000 deaths occurred (Siegel et al., 2020). Although tremendous research efforts have been made in early diagnosis, and the development of novel therapeutic strategies, the worldwide mortalities of breast cancer are still increasing every year. For breast cancer treatment, the most promising immunotherapy is the immune checkpoint blockage by anti-PD-1/PD-L antibodies, which achieves an objective response rate between 12 and 21% (Adams et al., 2019). However, the response to single-agent activity is restricted to less than 10% of patients with metastasis (Emens, 2018). Therefore, it is necessary to identify effective molecular targets and develop combination therapy for better treatment outcome.

CD4^+^Foxp3^+^ regulatory T cells (Tregs) play indispensable roles in the prevention of autoimmune diseases (Sakaguchi et al., 2001; Shevach et al., 2006) and chronic inflammatory diseases (Coombes et al., 2005; Xystrakis et al., 2006). However, cancer cells can escape immune attack by recruiting and accumulating Tregs in tumor tissues. The established immunosuppressive tumor microenvironment (TME) therefore suppresses the function of effector T (Teff) cells and promotes tumor progression (Woo et al., 2001). Therefore, selectively targeting tumor-infiltrating Tregs and stimulating CD8^+^ Teff cells in TME are attractive strategies to boost cancer immunotherapy.

Tumor necrosis factor (TNF) has been reported to play a key role in modulating Treg activity (Valencia et al., 2006; Baron et al., 2007). One mechanism is that TNFs could bind to TNF receptors [such as tumor necrosis factor receptor 2 (TNFR2)] (Santee and Owen-Schaub, 1996) and activate the downstream PI3K/Akt or NF-κB pathway to promote the proliferation of Tregs (Rodríguez et al., 2011; Vanamee and Faustman, 2017). High expression of TNFs and their oncogenic roles have been reported in several types of cancers, including colon cancer, multiple myeloma, and renal cell carcinoma (Uhlén et al., 2005; Hamilton et al., 2011; Rauert et al., 2011; Nakayama et al., 2014; Ungewickell et al., 2015). In contrast to the widely expressed receptor TNFR1, TNFR2 is preferentially expressed on Tregs, and a high level of TNFR2 expression is associated with the enhanced suppressive activity of Tregs in human and mice (Chen et al., 2008, 2010, 2013). Since TNFR2 is highly expressed in tumor infiltrating Tregs and tumor cells (Chang et al., 2015; Chen and Oppenheim, 2017; Zhao et al., 2017), TNFR2 antagonist or other agents blocking TNFR2 expression could exert an antitumor effect. A previous study demonstrated that targeting TNFR2 with antagonistic antibodies inhibits the proliferation of ovarian cancer cells and tumor-associated Tregs, implying the therapeutic potential of anti-TNFR2 antibody in tumor immunotherapy (Torrey et al., 2017). Although previous studies reveal that TNFR2 is the main TNFR expressed by B and T lymphocytes in breast cancer draining lymph nodes (dLNs) (Ghods et al., 2019), and TNFR2 is implicated in the progression and prognosis of breast cancer (Yang et al., 2017), there is no direct evidence that targeting TNFR2 can benefit breast cancer treatment.

In this study, we first validated the upregulation of TNFR2 in breast cancer tissues and cell lines, which was also associated with a poor prognosis in breast cancer patient. We then explored the therapeutic potential of anti-TNFR2 antagonistic antibody in a breast cancer model. Anti-TNFR2 antibody impaired the proliferation and suppressive activity of Tregs. Anti-TNFR2 antibody treatment alone could significantly inhibit the tumorigenesis of murine mammary carcinoma 4T1 cells *in vivo*. The application of anti-TNFR2 antibody together with anti-PD-L1 treatment showed superior antitumor effect as compared with the monotherapy. In summary, our data suggest that anti-TNFR2 antibody could boost the effect of anti-PD-L1 therapy, which could be formulated as a novelty combination therapy for breast cancer.

## Materials and Methods

### Patient Samples

We collected 30 pairs of breast cancer tumor tissues and adjacent normal tissues from patients who underwent surgical removal in the Hubei Cancer Hospital from January 2017 to December 2019. None of the selected patients had received chemotherapy or radiation therapy prior to surgery. This study was approved by the Research Ethics Committee at the Hubei Cancer Hospital. All patients signed the informed consent for the approval of using their tissue samples in the study.

### Cell Culture

Human breast cancer cell lines of MCF7, BT549, BT474, MDA-MB-453, normal human mammary epithelial cell line MCF-10A, murine mammary epithelial cells HC11, murine breast cancer cell line 4T1, and murine colon cancer tumor cells CT26 were purchased from American Type Culture Collection (ATCC). Cells were cultured according to the manufacturer’s instructions in a humidified cell incubator with 5% CO_2_ at 37°C.

### Mouse and Therapeutic Antibody

Female wild-type BALB/c mice (6–8 weeks old) were purchased from Shanghai SLAC Laboratory (Shanghai, China). The animal study was approved by the Ethics Committee of Animal Research at the Hubei Cancer Hospital. InVivoMAb anti-mouse TNFR2 antibody (Clone TR75-54.7) and anti-mouse PD-L1 (B7H1) antibody (Clone 10F.9G2) were both purchased from Bio X Cell (Lebanon, NH, United States). InVivoMAb polyclonal Armenian hamster IgG (BE0091) was used as isotype control for anti-mouse TNFR2 antibody.

### RNA Extraction and qRT-PCR

Total RNA from tumor tissues or cells was extracted using Qiagen RNeasy Mini Kit (Qiagen, Hilden, Germany), and then cDNA was synthesized using Qiagen RT (Adams et al., 2019). First Strand Kit (Qiagen, Hilden, Germany). The resulting cDNA was diluted and analyzed in a 7500 Real Time PCR System (Applied Biosystems/Life Technologies, Carlsbad, CA, United States) using SYBR Premix EX TAQ II kit (RR820A, Takara, Dalian, China). The PCR cycling condition used is as follows: hot start for 10 min at 95°C; and amplification for 40 cycles at 95°C for 15 s, 55°C for 30 s, and 72°C for 30 s, with signal detection at the end of each cycle. Finally, the 2^–ΔΔ*Ct*^ method was used to analyze the relative expression level, and GAPDH was used as the internal reference gene. Primers for qPCR analysis were as follows:

hTNFR2-Forward: 5′-TGGGCCAAGTTCCTCTAGTG-3′, Re verse: 5′-CAGGTCACAGAGAGTCAGGG-3′; IL10 Forward: 5′-GCCGGGAAGACAATAACTGC-3′, Reverse: 5′-CTGGGG CATCACTTCTACCA-3′; IL17a Forward: 5′-GTCCAAACACT GAGGCCAAG-3′, Reverse: 5′-AGCTTCCCAGATCACAGAG G-3′; CXCL10 Forward: 5′-CGATGGATGGACAGCAGAGA-3′, Reverse: 5′-GGACTCAGACCAGCCCTTAA-3′; TNFα For ward: 5′-AAGTCAACCTCCTCTCTGCC-3′, Reverse: 5′-TGGATGAACACCCATTCCCT-3′; TGF-β1 Forward: 5′-AGGT CCTTGCCCTCTACAAC-3′, Reverse: 5′-TCCTTAAATACA GCCCCGGG-3′; IFN-γ Forward: 5′-CGGCTGACCTAG AGAAGACA-3′, Reverse: 5′-TTTCAATGACTGTGCCGTGG-3′; and mTNFR2 Forward: 5′-ACACCCAGCCAAGTAGACTC-3′, Reverse: 5′-CAAATTGTTCCCTGCTCCCC-3′. GAPDH Forward: 5′-AGGTCGGTGTGAACGGATTTG-3′, Reverse: 5′-TGTAGACCATGTAGTTGAGGTCA-3′.

### Immunohistochemistry

For all immunohistochemistry (IHC) staining, 4-μm formalin-fixed, paraffin-embedded (FFPE) tissue sections on positively charged slides were used. FFPE sections were deparaffinized on a Prisma autostainer (Sakura, Torrance, CA, United States) and then subjected to antigen retrieval at high temperature (98°C) with high pH (9.0) Tris-EDTA buffer (Dako, Carpinteria, CA, United States) using a Lab Vision PT Module (Thermo Fisher Scientific, Carlsbad, CA, United States). Following antigen retrieval, sections were blocked with 5% normal goat serum for 3 h and then stained with anti-TNFR2 primary antibody overnight (InVivoMAb anti-mouse TNFR2 antibody (Clone TR75-54.7), dilution: 1:500). Staining was performed using the Autostainer 360 (Thermo Fisher Scientific, Waltham, MA, United States). Then antibody solution was removed, and the section was washed three times using TBST buffer for 5 min each. Slides were blocked for both endogenous peroxidase and non-specific protein binding by using Dual Endogenous Enzyme Block (Dako, Carpinteria, CA, United States) and UV blocking reagent (Thermo Fisher Scientific, Waltham, MA, United States). The section was soaked with one to three drops of SignalStain^®^ Boost Detection Reagent [horseradish peroxidase (HRP); Rabbit #8114, Cell Signaling Technology, Danvers, MA, United States] and incubated in a humidified chamber for 30 min at room temperature; 100–400 μl of SignalStain^®^ substrate (#8059, Cell Signaling Technology) was added to each section for 5 min. The section was washed in dH_2_O two times for 5 min each and then dehydrated. The section was mounted with coverslips using the mounting medium (#14177, Cell Signaling Technology) and photographed by light microscope (Olympus, Shinjuku, Tokyo, Japan).

### Primary Cell Isolation

Mouse CD4^+^ T cells and CD4^+^CD25^+^ Tregs were purified from pooled spleen and lymph nodes (including inguinal, axillary, and mesenteric regions) of wild-type C57BL/6 or BALB/c mice, using the mouse CD4^+^ T cell Isolation Kit (#130-095-248) or CD4^+^CD25^+^ Regulatory T Cell Isolation Kit (Cat #130091041; Miltenyi Biotec, Bergisch Gladbach, Germany). The purity of CD4^+^Foxp3^+^ Tregs achieved by CD4^+^CD25^+^ Regulatory T Cell Isolation Kit is ∼90% as previously reported (Gangi et al., 2005). The purified T cells were cultured in a round-bottom 96-well plate with RPMI-1640 medium (Corning, Christiansburg, VA, United States) containing 10% heat-inactivated fetal bovine serum (FBS; BioWhittaker, Walkersville, MD, United States), 2 mM of L-glutamine, 10 mM of HEPES buffer, 0.1 mM of non-essential amino acids, 1 mM of sodium pyruvate, 1% penicillin (100 U/ml)/streptomycin (100 mg/ml), and 50 μM of 2-methylmercaptoethanol. To maintain the survival and activation of CD4^+^CD25^+^ T cells, the plate was precoated with 5 μg/ml of anti-CD3 antibody, and the culture medium was supplied with 2 μg/ml of soluble anti-CD28 antibody and 10 ng/ml of IL2.

### Cell Counting Kit-8 Cell Proliferation Assay

To examine the proliferation, cells were seeded into a 96-well plate at the density of 2,000 cells/well and treated with isotype control IgG and increasing doses of anti-TNFR2 antibody (1, 5, 10, 25, and 50 μg/ml). After 1-, 2-, 3-, and 4-day posttreatment, cell proliferation was detected using a Cell Counting Kit-8 (CCK-8; Dojindo, Japan) following the manual instructions, the absorbance was measured at 450 nm using FLUOstar Omega (BMG Labtech, Ortenberg, Germany).

### Fluorescence-Activated Cell Sorting Staining and Apoptosis Assay

For apoptosis assay, cells were seeded in a 24-well plate at 50,000 cells/well. Cells were treated by isotype control IgG and anti-TNFR2 antibody for 48 h and then stained with 25 μg/ml of Annexin V and 10 μg/ml of propidium iodide (PI) for 15 min at 25°C in the dark. Subsequently, cell apoptosis was measured using LSR II (BD FACSCalibur, BD Biosciences, San Jose, CA, United States) flow cytometer.

For cell staining, phycoerythrin (PE) anti-human CD120b (TNFR2) antibody was purchased from BioLegend (San Diego, CA, United States); APC anti-mouse Foxp3 (FJK-16s) for intracellular staining of Foxp3 was purchased from Invitrogen (Carlsbad, CA, United States). Percp/cy5.5 anti-mouse CD45, fluorescein isothiocyanate (FITC) anti-mouse CD4, APC anti-mouse CD8a, and PE anti-mouse CD120b (TNF R Type II/p75) antibody (clone TR75-89) were purchased from BioLegend (United States). Cells were resuspended in fluorescence-activated cell sorting (FACS) buffer [phosphate-buffered saline (PBS) + 2% FBS + 0.05% NaN_3_]; cell surface staining was conducted at room temperature for 30 min. For intracellular staining of Foxp3, cells were fixed and permeabilized using Transcription Factor Staining Buffer Kit (Cat #TND-0607-KIT, TONBO Biosciences, San Diego, CA, United States). Then the cells were washed twice with 1 × Dulbecco’s PBS (DPBS) (Corning, VA, United States) and analyzed on LSR II (BD FACSCalibur). Flow cytometry data were analyzed by FlowJo software (version 10.6.1).

### Western Blotting Assays

Tumor tissues were collected, cut into pieces, and then dissolved in radioimmunoprecipitation assay (RIPA) lysis buffer containing protease inhibitor cocktail (Invitrogen, United States). Protein concentration was quantified by a BCA Protein Assay Kit (Solarbio, Beijing, China). Ten micrograms of total protein extract was used for sodium dodecyl sulfate–polyacrylamide gel electrophoresis (SDS-PAGE) electrophoresis. Separated protein in SDS-PAGE gel was transferred onto polyvinylidene difluoride (PVDF) membrane (Bio-Rad Laboratories, Hercules, CA, United States). After being blocked with 5% skimmed milk for 1 h, the membrane was then incubated with primary antibodies overnight at 4°C: anti-IL10, anti-IL17a, anti-CXCL10, anti-IFN-γ, anti-TNFα, anti-TGF-β1, and anti-TNFR2 antibody (Cell Signaling Technology, MA, United States). The membrane was washed three times with TBST for 5 min each. After being washed, the membrane was further incubated with HRP-linked secondary antibody (1:3,000; #7074; Cell Signaling, MA, United States) at room temperature for 1 h. Then the membrane was washed four times with 1 × TBST, and the protein bands were visualized using an enhanced Chemiluminescence Kit (Santa Cruz Biotechnology, Dallas, TX, United States) and photographed on a gel imager system (Bio-Rad, CA, United States).

### Effects of Anti-Tumor Necrosis Factor Receptor 2 on Regulatory T Cells

CD4^+^CD25^+^ Tregs were seeded in a round-bottom 96-well plate at a density of 0.2 × 10^6^ cells/well. The plate was precoated with added 5 μg/ml of plate-bound anti-CD3 antibody, and the culture medium was supplied with 2 μg/ml of soluble anti-CD28 antibody and 10 ng/ml of IL2. After 24 h, cells were treated with isotype control IgG and increasing doses of anti-TNFR2 antibody (1, 5, 10, 25, and 50 g/ml) for 72 h. After incubation, cells were harvested and centrifuged, washed once with 1 × DPBS, and then stained with fluorophore conjugated antibodies (anti-mouse CD4, anti-mouse Foxp3, and anti-mouse TNFR2) for FACS analysis.

### *In vitro* Cell Proliferation and Suppressive Function Assay

To detect the proliferation of Tregs, purified CD4^+^CD25^+^ T cells were labeled with CellTrace carboxyfluorescein succinimidyl ester (CFSE) cell proliferation reagent (Thermo Fisher Scientific, Waltham, MA, United States) according to the manufacturer’s instructions. After the treatment with anti-TNFR2 antibody, FACS analysis was performed to detect the proliferation of Tregs by quantifying the cells with diluted CFSE labeling.

To examine the suppressive function of Tregs on Teff cells, MACS-purified CD4^+^CD25^+^ T cells were pretreated with anti-TNFR2 antibody for 2 days and then cocultured with CFSE-labeled CD4^+^CD25^–^ Teff cells at a desired ratio in the presence of 2 × 10^5^ irradiated APCs/well in a plate precoated with 5 μg/ml of anti-mouse CD3 antibody. After 48-h culture, the proliferation of CFSE-labeled Teff cells was determined by FACS.

### *In vivo* Antitumor Efficacy in Murine Breast Cancer Model

Twenty-four BALB/c mice were randomly divided into four groups (*n* = 6 in each group). All of the mice were subcutaneously (s.c.) injected with 4T1 tumor cells suspended in PBS on the right flank closing to mammary fat pads (2 × 10^5^ 4T1 cells in 100 μl of PBS). Once the tumors reached 100–200 mm^3^ (usually 7–10 days after tumor inoculation), the tumor-bearing mice were intraperitoneally (i.p.) injected with hamster IgG isotype (100 μg), anti-TNFR2 antagonistic antibody (100 μg), anti-PD-L1 (100 μg), or anti-TNFR2 antagonistic (100 μg) + anti-PD-L1 (100 μg) twice per week. The tumor size and body weight of tumor-bearing mice were monitored twice a week. Animals were euthanized when tumor volume reached 2,000 mm^3^, or large necrotic lesions were observed, or when 20% weight loss was observed.

To detect the development of 4T1 cell specific immunity, mice should survive the first round of 4T1 cell inoculation after anti-TNFR2 therapy, or combination therapy was rechallenged by subcutaneous (s.c.) injection with 4T1 cells (2 × 10^5^ cells/100 μl) and CT26 cells (2 × 10^5^ cells/100 μl) on contralateral flanks simultaneously. The growth of both tumor cells was monitored twice a week.

### Isolation of Tumor-Infiltrating Lymphocytes and Immunostaining

After antibody treatment, tumors and dLNs (inguinal lymph node at the same side of tumor injection) were surgically dissected and dissociated using MACS^®^ Tissue Dissociation Kits (Miltenyi Biotec, Bergisch Gladbach, Germany). After dissociation, red blood cells were lysed using 1 × ACK lysing buffer (Invitrogen, Thermo Fisher, United States). The remaining cells were washed twice using FACS buffer and centrifuged at 400 × *g* for 5 min. Cells were stained with corresponding antibodies, and FACS analysis was performed to quantify the changes of CD4^+^ T, CD8^+^ T, and Treg cells in the samples.

### Statistical Analysis

The statistical significance between groups was analyzed by paired Student’s *t*-test, and comparisons among multiple groups were analyzed using one-way analysis of variance (ANOVA) with Tukey’s *post hoc* test for pairwise comparison. Comparisons of data at multiple time points were examined using two-way ANOVA. Kaplan–Meier (KM) curve and log-rank test were used to compare the cumulative survival rates of different groups of mice. Statistical test was analyzed using GraphPad Prism version 8.0. The results were represented as mean ± standard deviation (SD) of multiple experiments. Statistical significance was determined by ^∗^*p* < 0.05, ^∗∗^*p* < 0.01, and ^∗∗∗^*p* < 0.001.

## Results

### Tumor Necrosis Factor Receptor 2 Is Upregulated in Breast Tumor Cells and Associated With a Poor Prognosis of Breast Cancer Patients

**FIGURE 1:**
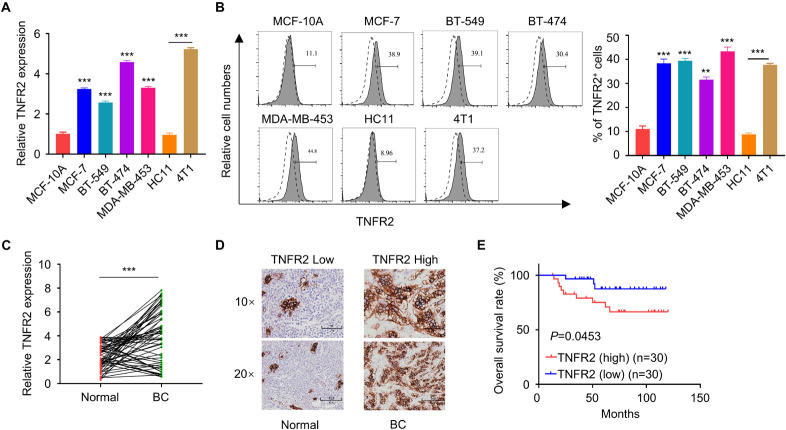
Tumor necrosis factor receptor 2 is overexpressed in breast tumor and correlated with a poorer overall survival of breast cancer patients. **(A)** qRT-PCR showed that *TNFR2* mRNA level was significantly higher in human breast cancer cell lines (MCF-7, BT549, BT474, and MDA-MB-453) than human mammary epithelial cell MCF-10A (***p < 0.001). Likewise, *TNFR2* mRNA level was higher in mouse breast cancer cell line (4T1) than mouse mammary epithelial cell HC11 (***p < 0.001). **(B)** Fluorescence-activated cell sorting (FACS) analysis of TNFR2 expression in human and mouse breast cancer cell lines. The results showed that TNFR2 was highly expressed in both human and mouse tumor cells lines as compared with normal epithelial cell lines (**p < 0.05, ***p < 0.001). Dotted line: TNFR2 antibody isotype IgG staining. Solid line: TNFR2 staining. **(C)** qRT-PCR analysis showed that compared with those in adjacent non-tumor tissues (*n* = 30), the mRNA levels of *TNFR2* were significantly higher in breast cancer tissues (*n* = 30, ***p < 0.001). **(D)** Immunohistochemistry (IHC) analysis showed a higher expression of TNFR2 in breast cancer tissues as compared with non-tumor tissues. **(E)** Overall survival analysis of TNFR2 high-expression group and TNFR2 low-expression group by Kaplan–Meier plot demonstrated a poorer prognosis in TNFR2 high-expression group (*p = 0.0453).

To validate the expression of TNFR2 in breast cancer cells, we first detected its mRNA and protein levels in normal human mammary epithelial cell line MCF-10A and a number of human breast cancer cell lines (MCF7, BT549, BT474, and MDA-MB-453), as well as in murine breast cancer cell line 4T1 and murine mammary epithelial cell HC11. Compared with MCF-10A cells, all human breast cancer cell lines showed a significant increase of TNFR2 at mRNA and protein levels. Similar results were observed in murine breast tumor cells of 4T1 as compared with the epithelial cell line HC11 (^∗∗∗^*p* < 0.001, Figures 1A,B). Moreover, we purified mRNA from 30 pairs of breast tumor tissues and adjacent normal tissues from breast cancer patients. qRT-PCR analysis showed that *TNFR2* mRNA level was also significantly higher in tumor tissues as compared with the paired para-tumoral tissues (^∗∗∗^*p* < 0.001, Figure 1C). As expected, in breast cancer tissues, a much stronger TNFR2 protein level was observed by IHC (Figure 1D). Then the patients were divided into high-expression and low-expression groups based on the medina value of TNFR2 expression in qRT-PCR analysis. KM plot analysis revealed that high expression of TNFR2 was associated with a poor prognosis in breast cancer patients (*p* = 0.0453, Figure 1E). Moreover, Chi-square test demonstrated that high TNFR2 expression was associated with a larger tumor size, more advanced TNM stage, and lymph node metastasis, while no significant correlation was observed with the patient’s age (Table 1).

**TABLE 1:** The clinical characteristics of breast cancer patients.

Factor		TNFR2 expression		*p*-Value
		Low (*n* = 30)	High (*n* = 30)	
Age				0.347
	≤60	22	25	
	>60	8	5	
Tumor size				0.009
	≤4 cm	18	8	
	>4 cm	12	22	
TNM stage				0.02
	I/II	20	11	
	III/IV	10	19	
Lymph node metastasis				0.009
	Negative	22	12	
	Positive	8	18	

*According to Figure 1D, the Chi-square test was used to calculate the relationship between TNFR2 expression and breast cancer clinicopathological data [high TNFR2 expression was correlated to a larger tumor size, more advanced TNM stage, more lymph node metastasis (p < 0.05). No significant correlation was observed with the patient’s age].*

### Anti-Tumor Necrosis Factor Receptor 2 Antibody Inhibits 4T1 Cell Proliferation and Promote Its Apoptosis

**FIGURE 2:**
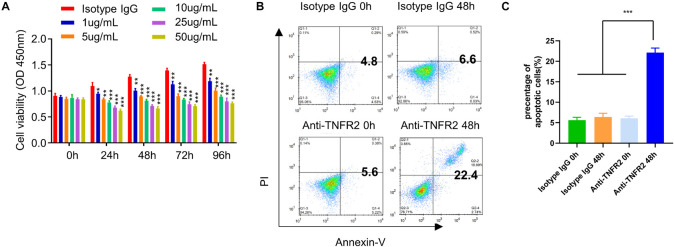
Anti-TNFR2 antibody inhibits murine breast cancer cell proliferation and induces cell apoptosis. **(A)** Murine breast tumor cell line 4T1 was treated with different concentrations of anti-TNFR2 antibody (1, 5, 10, 25, and 50 μg/ml) or the isotype IgG (50 μg/ml). Cell Counting Kit-8 (CCK-8) cell proliferation assay showed that anti-TNFR2 antibody could inhibit the tumor cell proliferation in a dose-dependent manner. **(B)** Annexin V/propidium iodide (PI) apoptosis assay by fluorescence-activated cell sorting (FACS). After 48-h treatment with anti-TNFR2 antibody (10 μg/ml), the apoptotic events of 4T1 tumor cells were significantly increased as compared with those of the isotype IgG treatment (10 μg/ml). The number in the FACS plot indicates the percentage of apoptotic cells in quadrat 2 and 4. **(C)** Summary of the proportion of apoptotic cells. Data are the summary of three independent experiments. *p < 0.05, **p < 0.01, ***p < 0.001.

Since TNFR2 was highly expressed on tumor cells, we next attempted to examine whether targeting TNFR2 affects the survival of tumor cells. We used a monoclonal antibody, which blocks ligand-induced receptor signaling of TNFR2 *in vitro* and *in vivo* (DeBerge et al., 2015; Leclerc et al., 2016). After the treatment with increasing dose of anti-TNFR2 antibody (1, 5, 10, 25, and 50 μg/ml), CCK-8 cell proliferation assay showed that anti-TNFR2 antibody could inhibit the proliferation of mouse breast tumor cell line 4T1 in a dose-dependent manner (Figure 2A). We also performed apoptosis assay after 48 h treatment with isotype IgG and anti-TNFR2 antibody. FACS analysis demonstrated that anti-TNFR2 antibody (10 μg/ml) significantly enhanced the percentage of apoptotic events as compared with cells treated with isotype control IgG (^∗∗∗^*p* < 0.001, Figures 2B,C).

### Anti-Tumor Necrosis Factor Receptor 2 Affects Foxp3 Expression in Regulatory T Cells and Reduces the Inhibitory Effect of Regulatory T Cells on Effector T Cells

**FIGURE 3:**
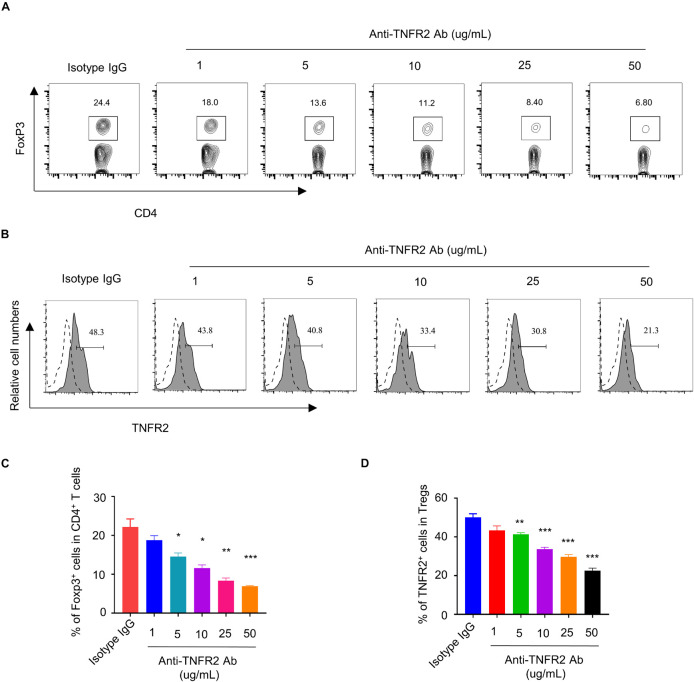
Anti-TNFR2 antibody decreases the percentage of regulatory T cells (Tregs) in CD4^+^ T-cell population and downregulates TNFR2 expression. **(A)** Fluorescence-activated cell sorting (FACS) analysis of Foxp3 intracellular staining in CD4^+^ T cells after anti-TNFR2 antibody treatment. **(B)** FACS analysis of TNFR2 expression in CD4^+^Foxp3^+^ Tregs after anti-TNFR2 antibody treatment. Dotted line: TNFR2 antibody isotype IgG staining. Solid line: TNFR2 staining. **(C)** Summarized data of the proportion of CD4^+^Foxp3^+^ Tregs in CD4^+^ T-cell population. **(D)** Summary of the percentage of TNFR2-positive cells. Data are the summary of three independent experiments. *p < 0.05, **p < 0.01, ***p < 0.001.

Tumor Necrosis Factor Receptor 2 was preferentially expressed by CD4^+^Foxp3^+^ Tregs and has been reported to stimulate the proliferation and activation of Tregs (Zhao et al., 2017). To demonstrate the effect of anti-TNFR2 antibody on Tregs, we hypothesized that anti-TNFR2 antibody could affect the proliferation of Tregs and the suppressive function on Teff cells. MACS-purified CD4^+^ T cells were treated with different doses of anti-TNFR2 antibody (1, 5, 10, 25, and 50 μg/ml) or isotype IgG for 3 days. Intracellular staining of Foxp3 showed that anti-TNFR2 antibody reduced the proportion of CD4^+^Foxp3^+^ Tregs in a dose-dependent manner in CD4^+^ T-cell population (Figures 3A,C). Meanwhile, anti-TNFR2 antibody treatment also significantly decreased the TNFR2 expression by Foxp3^+^ Tregs in a dose-dependent manner, as revealed by TNFR2 surface staining using a different antibody (PE anti-mouse CD120b (TNF R Type II/p75) antibody (clone TR75-89) (Figures 3B,D).

**FIGURE 4:**
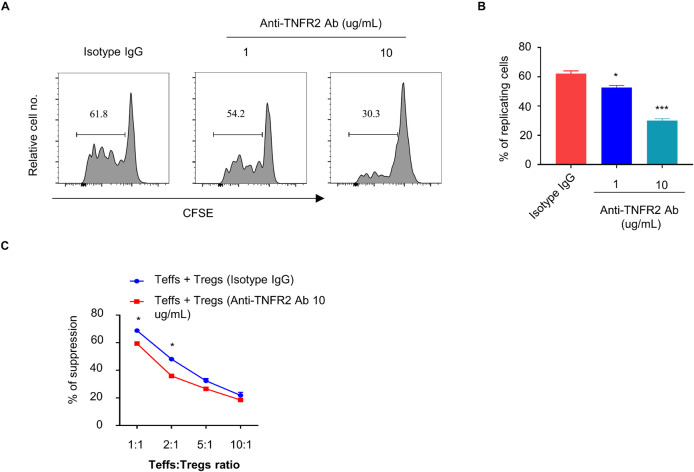
Anti-TNFR2 antibody reduces the proliferation and suppressive activity of regulatory T cells (Tregs). CD4^+^CD25^–^ T cells (Teffs) and CD4^+^Foxp3^+^ T cells (Tregs) from pooled spleen and lymph nodes (inguinal, axillary, and mesenteric regions) of BALB/c mice were purified. **(A)** Carboxyfluorescein succinimidyl ester (CFSE)-labeled CD4^+^Foxp3^+^ T cells (Tregs) were treated with anti-TNFR2 antibody (1 and 10 μg/ml) or isotype IgG (10 μg/ml) for 72 h. CFSE dilution analysis showed that anti-TNFR2 antibody inhibited the proliferation of Tregs. **(B)** Summary of the percentage of proliferating CD4^+^Foxp3^+^ Tregs with CFSE dilution from three independent experiments. **(C)** Tregs pretreated with anti-TNFR2 antibody (10 μg/ml) or isotype IgG (10 μg/ml) were cocultured with CFSE-labeled CD4^+^CD25^–^ Teffs at different ratios in the presence of irradiated APCs and 5 μg/ml of anti-CD3 antibody for 72 h. Anti-TNFR2 antibody pretreatment reduced the suppressive activity of Tregs. Data are the summary of three independent experiments. *p < 0.05, **p < 0.01, ***p < 0.001.

To further examine the effect of anti-TNFR2 antibody on the proliferation of Tregs, CFSE-labeled CD4^+^CD25^+^ Tregs were treated with isotype IgG or anti-TNFR2 antibody (1 and 10 μg/ml) for 3 days, and FACS analysis showed that anti-TNFR2 antibody significantly inhibited the proliferation of Tregs as indicated by the reduced number of cells showing CFSE dilution (Figures 4A,B). However, when anti-TNFR2 antibody was treated on CD4^+^CD25^–^ Teff cells, there was no remarkable suppression on Teff cells (data not shown). Furthermore, suppressive activity assay was performed by the coculture of Tregs (pretreatment with isotype IgG or anti-TNFR2 antibody) with CFSE-labeled Teffs (CD4^+^CD25^–^ T cells) at desired ratios (1:1, 1:2, 1:5, and 1:10) in the presence of irradiated APCs and anti-CD3 antibody. The results showed that anti-TNFR2 pretreatment in Tregs reduced the suppressive activity of Tregs on Teffs (^∗^*p* < 0.05, Figure 4C). Collectively, these results suggest that TNFR2 antagonistic antibody could negatively affect Treg proliferation and suppressive activity.

### Anti-Tumor Necrosis Factor Receptor 2 Therapy Promotes the Survival of Mice Bearing 4T1 Tumor and Develops 4T1-Specific Immune Response

**FIGURE 5:**
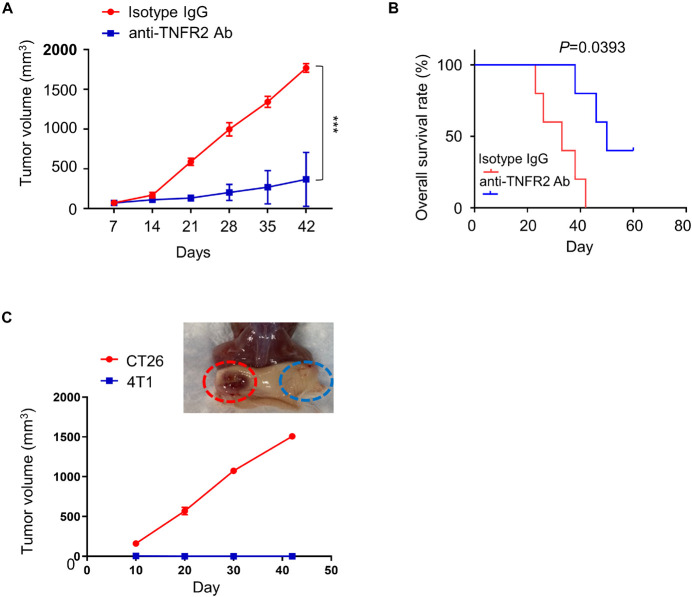
Anti-TNFR2 therapy inhibits 4T1 tumorigenesis and generates tumor specific immunity. Twelve BALB/c mice were randomly divided into two groups (six mice/group). All of the mice were subcutaneously (s.c.) injected with 4T1 tumor cells. Once the tumor was established after 1 week, the tumor-bearing mice were intraperitoneally injected with anti-TNFR2 antibody (100 μg/kg) or isotype IgG control every 3 days (twice per week). **(A)** Anti-TNFR2 therapy suppressed tumor growth as compared with control group, and two mice within anti-TNFR2 treatment reached complete response (***p < 0.001). **(B)** Kaplan–Meier plot analysis demonstrated that anti-TNFR2 treatment significantly increased the overall survival when compared with the isotype IgG control group mice (*p = 0.0393). **(C)** Two cured mice from anti-TNFR2 treatment were inoculated with 4T1 (2 × 10^5^ cells/100 μl) and CT26 (2 × 10^5^ cells/100 μl) on contralateral flanks. The tumor growth was monitored, and representative tumor formation image is shown. Tumor growth of 4T1 cells was totally inhibited, while CT26 tumor continued growing.

To evaluate the *in vivo* therapeutic potential of anti-TNFR2 therapy, BALB/c mice were subcutaneously injected with 4T1 tumor cells on the right flank, and once tumor size reached 50–100 mm^3^, animals were randomly divided into two groups (*n* = 6 in each group): treatment group with anti-TNFR2 antibody intraperitoneally (i.p.) injection every 3 days and control group with IgG isotype intraperitoneally (i.p.) injection every 3 days. We observed that anti-TNFR2 treatment showed a strong antitumor effect by significantly suppressing tumor growth as compared with isotype IgG control, and two mice in the treatment group reached complete response (CR) (Figure 5A). Moreover, the overall survival of tumor-bearing mice was also significantly prolonged by anti-TNFR2 treatment (*p* = 0.0393, Figure 5B).

To explore whether the cured tumor-free mice acquired 4T1-specific immunity, the two mice reached CR after anti-TNFR2 treatment was subcutaneously injected with either 4T1 or CT26 tumor cells on contralateral flanks, and the tumor formation of both flanks was monitored twice per week. We observed that the tumor volume of CT26 cell continued to increase, whereas none of the mice developed visible 4T1 tumor (Figure 5C). Together, these results indicate that anti-TNFR2 treatment has a protective effect against the tumorigenesis of breast cancer cell and could lead to tumor-specific immunity.

### Anti-Tumor Necrosis Factor Receptor 2 Antibody Combined With Anti-PD-L1 Therapy Shows Synergistic Antitumor Effect

**FIGURE 6:**
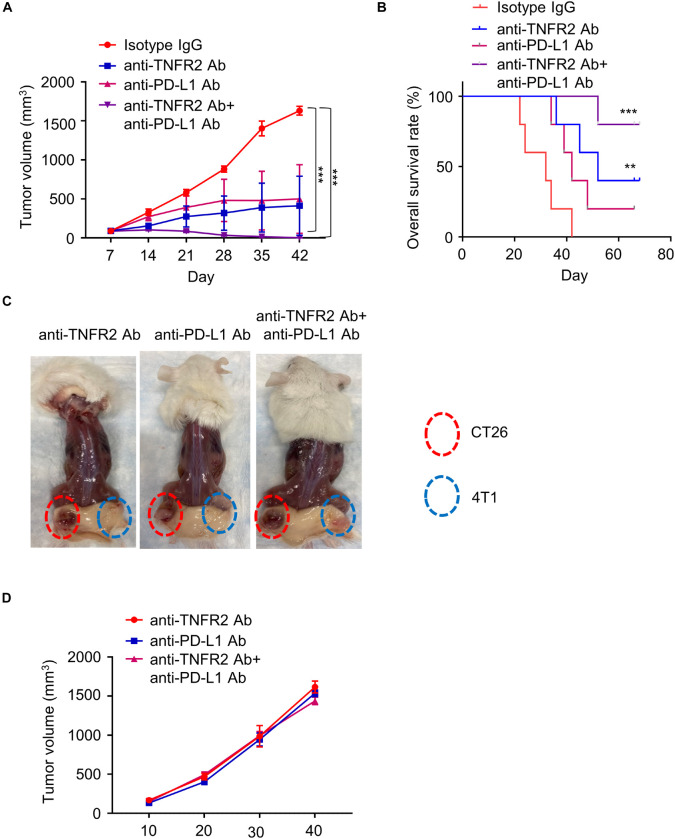
Anti-TNFR2 antibody in combination with anti-PD-L1 therapy exerts superior antitumor effect than monotherapy. Twenty-four BALB/c mice were inoculated with 4T1 cells, and the tumor-bearing mice were randomly divided into four groups (six mice/group): isotype IgG control group, anti-TNFR2 group, anti-PD-L1 group, and anti-TNFR2^+^ anti-PD-L1 combination group (two doses per week). **(A)** Anti-TNFR2 antibody in combination with anti-PD-L1 therapy largely suppressed tumor growth. When compared with control group, anti-TNFR2 antibody monotherapy and combination therapy significantly inhibited the increase of tumor volume, with the combination therapy showing stronger effect (***p < 0.001). **(B)** Kaplan–Meier plot shows that combination therapy significantly improved the overall survival (**p < 0.01, ***p < 0.001, four mice showed complete response in combination therapy). **(C)** The tumor-free mice after monotherapy and combination therapy were rechallenged with 4T1 (2 × 10^5^ cells/100 μl) and CT26 (2 × 10^5^ cells/100 μl) cells on contralateral flanks. Representative photographs from each group demonstrated that tumor growth of 4T1 cells was totally inhibited while CT26 tumor continued growing. **(D)** The tumor growth curve of CT26 cells in tumor-free mice after mono- or combination therapy. No difference was observed between mice after monotherapy and combination therapy.

To evaluate whether the application of anti-TNFR2 antibody enhanced the effect of immune checkpoint blockage of anti-PD-L1 therapy, 24 BALB/c mice were subcutaneously injected with 4T1 tumor cells and randomly divided into four groups (six mice/group). Mice were intraperitoneally injected with isotype IgG, anti-TNFR2 antibody, anti-PD-L1 antibody, or both antibodies for a total of five doses. Although one out of six mice in anti-PD-L1 monotherapy reached CR, four mice in the group receiving combination therapy of anti-TNFR2 with anti-PD-L1 reached CR, and the tumor growth was significantly suppressed (Figure 6A). KM plotter analysis indicated anti-TNFR2 combined with anti-PD-L1 therapy could significantly increase the overall survival when compared with the monotherapy group (Figure 6B). The tumor-free mice were rechallenged with 4T1 and CT26 tumor cells. As predicted, all these mice developed CT26 tumor without discernible difference, whereas 4T1 tumorigenesis was not observed (Figures 6C,D).

### Anti-Tumor Necrosis Factor Receptor 2 Therapy and Combinatory Therapy Changes the Immune Profiles in Tumor Tissues

**FIGURE 7:**
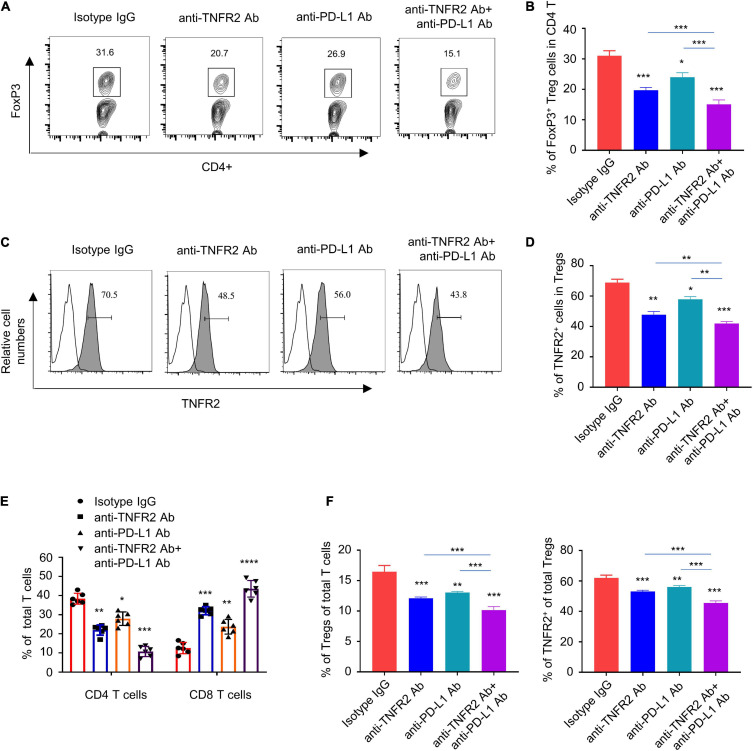
Anti-TNFR2 treatment or combination therapy reduces tumor-infiltrating regulatory T cells (Tregs) and increased the percentage of CD8 effector T cells. Tumor tissue and the tumor-associated draining lymph nodes of each group after three doses of mono- or combination therapy (*n* = 6 mice in each group) were collected. **(A)** Fluorescence-activated cell sorting (FACS) analysis of intracellular Foxp3 staining showed that anti-TNFR2 antibody in combination with anti-PD-L1 treatment greatly reduced the proportion of regulatory T cells (Tregs) in total CD4^+^ T cells in tumor tissue (***p < 0.001). **(B)** Summary of the proportion of CD4^+^Foxp3^+^ Tregs in total CD4^+^ T cells in **(A)**. **(C)** FACS analysis showed the decreased expression of TNFR2 in Tregs after monotherapy and combination therapy (blank line: isotype IgG staining; line with shadow: TNFR2 staining; ***p < 0.001). **(D)** Summary of the percentage of TNFR2 expressing Tregs shown by histogram. **(E)** Anti-TNFR2 antibody therapy and combination therapy decreased the proportion of CD4^+^ T cell, while they increased the proportion of CD8^+^ T cells in total T-cell population (***p < 0.001). **(F)** FACS analysis in tumor-associated draining lymph nodes showed that combination therapy significantly reduced the proportion of CD4^+^Foxp3^+^ Tregs in total CD4^+^ T cells and decreased the expression of TNFR2 in Tregs. *p < 0.05, **p < 0.01, ***p < 0.001.

To examine the changes of tumor-infiltrating lymphocytes and immune cells in tumor dLNs, we harvested tumor tissue and the dLNs of all tumor-bearing mice after three doses of treatment with isotype IgG, anti-TNFR2, anti-PD-L1, or the combination. Anti-TNFR2 monotherapy and combinatory therapy significantly reduced the percentage of Foxp3^+^ Tregs in CD4^+^ T cells in tumor tissue, with the combination therapy showing a stronger effect (Figures 7A,B). In addition, the expression level of TNFR2 in Foxp3^+^ Tregs was also decreased by both monotherapy and combinatory therapy (Figures 7C,D). Furthermore, anti-TNFR2 antibody alone or the combinatory therapy decreased the proportion of CD4^+^ T cell in total T cells, while the proportion of CD8^+^ T cells was significantly increased (Figure 7E). In tumor tissues and the dLNs, anti-TNFR2 monotherapy and combinatory therapy also significantly reduced the proportion of Foxp3^+^ Tregs in total T cells and decreased TNFR2 expression, with the combinatory therapy showing an augmented effect (Figure 7F). Together, these results indicate that anti-TNFR2 treatment can boost immune checkpoint blockage by negatively affecting Tregs in tumor tissues.

### Anti-Tumor Necrosis Factor Receptor 2 Therapy and Combinatory Therapy Modulate the Cytokine Profiles in Tumor Tissues

**FIGURE 8:**
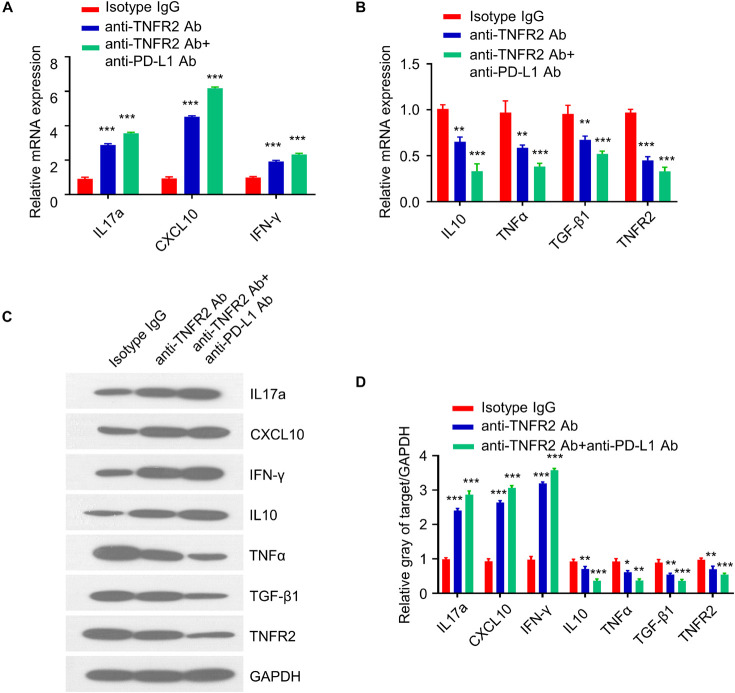
Anti-TNFR2 therapy and combinatory therapy modulate the cytokine profiles in tumor tissues. Total RNA was purified from breast tumor tissues (*n* = 6 mice in each group) after three doses of treatment of monotherapy and combination therapy. qRT-PCR and western blotting were performed to detect expression of proinflammatory and anti-inflammatory cytokines including IL17a, CXCL10, TNFα, TGF-β1, IFN-γ, and TNFR2. **(A)** Anti-TNFR2 antibody monotherapy and combination therapy increased the expression of IL17a, CXCL10, and IFN-γ. **(B)** qRT-PCR showed that the expression of IL10, TNFα, TGF-β1, and TNFR2 were downregulated after monotherapy and combination therapy, with combination therapy showing a stronger effect. **(C)** Western blotting analysis showed the upregulation of IL17a, CXCL10, and IFN-γ and the downregulation of IL10, TNFα, TGF-β1, and TNFR2 at protein level after monotherapy and combination therapy, with combination therapy showing a stronger effect. **(D)** Densitometry analysis of the relative protein expression of different cytokines. Data were normalized to the level of GAPDH. Data are the summary of three independent experiments. *p < 0.05, **p < 0.01, ***p < 0.001.

Since the antitumor effect is mediated by immunostimulatory or immunosuppressive cytokines, we next examined the expression of pro- and anti-inflammatory cytokines in tumor tissues after three doses of treatment. qRT-PCR analysis showed that anti-TNFR2 therapy and combinatory therapy significantly increased the expression of IL17a, CXCL10, and IFNγ as compared with the isotype control (Figure 8A), while the expression of IL10, TNFα, TGF-β1, and TNFR2 was downregulated (Figure 8B). These results were further validated at the protein level by western blotting. Western blotting results demonstrated the upregulation of IL17a, CXCL10, and IFNγ and the downregulation of TNFα, TGF-β1, and TNFR2 after anti-TNFR2 therapy and combinatory therapy (Figures 8C,D). These data indicate that anti-TNFR2 treatment and anti-TNFR2 in combination with anti-PD-L1 therapy could increase the level of proinflammatory cytokines such as IFN-γ and IL17a but decrease the anti-inflammatory cytokine such as IL10 and TGF-β1.

## Discussion

Cancer immunotherapy has revolutionized the treatment of human cancers. However, due to the insufficient infiltration of Teff cells and the accumulation of immunosuppressive Tregs in TME, immune evasion by tumor cells seriously hinders the efficacy of immunotherapy (Farkona et al., 2016; Waldman et al., 2020). TNFR2 has been implicated in promoting the progression of different types of tumors such as breast cancer (Rivas et al., 2008) and colon cancer (Yang et al., 2015; Torrey et al., 2017), as well as driving the proliferation of tumor stem cells and accelerating metastasis in renal cancer (Al-Lamki et al., 2010, 2016). Previous studies reported that not only the effect of TNFR2 is restricted in tumor cells, but also TNFR2 regulates the function of T lymphocytes, especially Tregs. Tregs are key players in establishing the immunosuppression in TME (Byrne et al., 2011; Facciabene et al., 2012; Cari et al., 2018), and TNFR2 is highly expressed in a subset of Tregs with maximal suppressive activity (Chen et al., 2007, 2008, 2010; Nguyen and Ehrenstein, 2016; Vanamee and Faustman, 2017). Previous studies indicate that the high expression level of TNFR2 could maintain the activity of tumor-infiltrating Tregs in melanoma (Chopra et al., 2013) and promote the expansion of Tregs in liver cancer and ovarian cancer (Chang et al., 2015; Chen and Oppenheim, 2017). However, whether targeting TNFR2 in breast cancer could also exert an antitumor effect by negatively affecting Tregs remains to be elucidated.

In this study, we demonstrated that TNFR2 was highly expressed in breast tumor tissues and cells. The high expression level of TNFR2 is also associated with a poor prognosis of breast cancer patients, indicating that high TNFR2 expression favors the progression of breast cancer. Our *in vitro* and *in vivo* study using TNFR2 antagonistic antibody demonstrated that targeting TNFR2 exerts an antitumor effect against breast cancer cell proliferation and induces tumor cell apoptosis. Although the molecular mechanism by which anti-TNFR2 antibody affects tumor cells is unclear, previous studies suggest that TNFR2 signaling promotes the cell proliferation by activating PI3K/Akt or NF-κB signaling (Rodríguez et al., 2011; Okubo et al., 2013; Chang et al., 2015). It is likely that the inhibitory effect of anti-TNFR2 antibody may result from the blockage of TNFR2 downstream signaling pathways.

As reported, TNFR2 is highly expressed by CD4^+^Foxp3^+^ Tregs and is associated with the immunosuppressive activity of tumor-infiltrating Tregs (Chen et al., 2007; Nguyen and Ehrenstein, 2016). We found that TNFR2 antagonist antibody could diminish the proportion of TNFR2^+^ Tregs in tumor tissues and induce the expansion of CD8^+^ Teff cells. The therapy by TNFR2 antibody improves the survival of tumor-bearing mice and leads to the establishment of tumor-specific immunity response. These data suggest that targeting TNFR2 could be an attractive strategy in breast cancer by negatively regulating tumor-infiltrating Tregs.

Previous studies indicate that the impaired efficacy of immune checkpoint inhibitor (ICI) therapy might be caused by the upregulation of TNFR2 in tumor-infiltrating Tregs (Chen et al., 2008; Tirosh et al., 2016; Williams et al., 2016). Our study provides evidence that TNFR2 antagonistic antibody can enhance the antitumor immunity in combination with anti-PD-L1 treatment in breast cancer by negatively affecting the accumulation of Tregs and increasing the percentage of Teff cells in tumor tissues. Furthermore, anti-TNFR2 monotherapy or in combination with anti-PD-L1 ICI therapy increases the expression of proinflammatory cytokines such as IFN-γ and IL17a but downregulates the anti-inflammatory cytokine such as IL10 and TGF-β1. These results support the notion that targeting TNFR2 could reshape the immune microenvironment in tumor tissues.

TNFR2 is overexpressed in a variety of cancers, and TNFR2-expressing Tregs are preferentially accumulated in TME. TNFR2 antagonist is emerging as an attractive candidate in cancer immunotherapy in recent years. In this study, we showed that anti-TNFR2 antibody combined with anti-PD-L1 therapy exerts a superior therapeutic effect in murine breast cancer model. TNFR2 antagonist antibody diminishes the proportion of Tregs but increases the percentage of Teff cells in tumor tissues. Our results imply that targeting TNFR2 could be applied together with ICI to produce profound therapeutic outcome in breast cancer treatment.

## Data Availability Statement

The raw data supporting the conclusions of this article will be made available by the authors, without undue reservation.

## Ethics Statement

The studies involving human participants were reviewed and approved by the Research Ethics Committee at the Hubei Cancer Hospital. The ethics committee waived the requirement of written informed consent for participation. The animal study was reviewed and approved by the Ethics Committee of Animal Research at the Hubei Cancer Hospital.

## Author Contributions

QF and WZ played a guiding role in the experimental design and data analysis. QS and JT reperformed the flow cytometry, handled the revision, edited the manuscript, and mainly took charge of writing. LH and YC revised the manuscript. All authors contributed to the article and approved the submitted version.

## Conflict of Interest

The authors declare that the research was conducted in the absence of any commercial or financial relationships that could be construed as a potential conflict of interest.

## Publisher’s Note

All claims expressed in this article are solely those of the authors and do not necessarily represent those of their affiliated organizations, or those of the publisher, the editors and the reviewers. Any product that may be evaluated in this article, or claim that may be made by its manufacturer, is not guaranteed or endorsed by the publisher.
